# Efficient framework for brain tumor detection using different deep learning techniques

**DOI:** 10.3389/fpubh.2022.959667

**Published:** 2022-12-01

**Authors:** Fatma Taher, Mohamed R. Shoaib, Heba M. Emara, Khaled M. Abdelwahab, Fathi E. Abd El-Samie, Mohammad T. Haweel

**Affiliations:** ^1^College of Technological Innovative, Zayed University, Abu Dhabi, United Arab Emirates; ^2^Faculty of Electronic Engineering, Menoufia University, Menouf, Egypt; ^3^Department of Information Technology, College of Computer and Information Sciences, Princess Nourah Bint Abdulrahman University, Riyadh, Saudi Arabia; ^4^Department of Electrical Engineering, Shaqra University, Shaqraa, Saudi Arabia

**Keywords:** MRI, CNN, segmentation, classification, brain tumor classification, deep neural networks, pre-trained models, transfer learning

## Abstract

The brain tumor is an urgent malignancy caused by unregulated cell division. Tumors are classified using a biopsy, which is normally performed after the final brain surgery. Deep learning technology advancements have assisted the health professionals in medical imaging for the medical diagnosis of several symptoms. In this paper, transfer-learning-based models in addition to a Convolutional Neural Network (CNN) called BRAIN-TUMOR-net trained from scratch are introduced to classify brain magnetic resonance images into tumor or normal cases. A comparison between the pre-trained InceptionResNetv2, Inceptionv3, and ResNet50 models and the proposed BRAIN-TUMOR-net is introduced. The performance of the proposed model is tested on three publicly available Magnetic Resonance Imaging (MRI) datasets. The simulation results show that the BRAIN-TUMOR-net achieves the highest accuracy compared to other models. It achieves 100%, 97%, and 84.78% accuracy levels for three different MRI datasets. In addition, the *k*-fold cross-validation technique is used to allow robust classification. Moreover, three different unsupervised clustering techniques are utilized for segmentation.

## 1. Introduction

The terminology of “brain tumor” involves the growth of abnormal cells in brain tissues. It is a grouping or bulk of abnormal brain cells (1). The skull, which acts as a protective shield for the brain, is extremely rigid. Any growth inside such a confined place might be dangerous. Brain tumors are categorized as being malignant (cancerous) or benign (not cancerous). There are two types of brain cancer: primary and secondary. A primary brain tumor develops within the brain, where many brain tumors in their early stages are not hazardous. A secondary brain tumor, also known as a metastatic brain tumor, occurs when cancer cells move from another organ, such as the lung or breast, to the brain.

A primary brain or spinal cord tumor develops within the brain or spinal cord. Primary malignant tumors of the brain and spinal cord have been detected in 24,530 people in the United States (13,840 males and 10,690 females). The likelihood of developing this type of tumors within one's lifetime is less than 1%. Approximately, 85–90% of all early malignancies are brain tumors. According to Cancer Net (2), brain or central nervous system tumors were detected in about 3,460 children under the age of 15. Pressure or headache around the tumor, loss of balance, and problems with fine motor skills are all symptoms of brain tumors. According to Cancer Net (2), a pineal gland tumor can induce vision alterations such as loss of eyesight, double vision, and inability to gaze upward.

Several researchers compared Computed Tomography (CT) with MRI for brain tumor diagnosis. MRI is more sensitive but less specific (3). There is an ability with MRI to detect abnormalities that are undetected or just faintly visible on CT. When CT scans indicate only a hazy aura, MRI may be used to confirm the tumor exact scope and location. Additionally, MRI with superior contrast discrimination and the ability to record images at several levels can aid in pinpointing the precise site of the lesion with respect to important neuroanatomical structures. As a consequence, we propose a framework for brain tumor detection from MRI datasets with multiple deep learning models.

The main problem considered in this paper is the classification of different brain tumor cases from magnetic resonance images based on segmentation after a first classification stage. This paper is concerned with the utilization of CNN models with different learning strategies for brain tumor detection. First, we consider the transfer-learning-based approach for brain tumor detection from magnetic resonance images. Different pre-trained deep learning models, namely, InceptionResNetv2, Inceptionv3, and ResNet50 are considered and compared for the task of brain tumor detection from magnetic resonance images. The second approach is the training of a proposed CNN model called BRAIN-TUMOR-net from scratch. The classification gives a decision about the case, whether anomalous or not. After that, the suspicious area is segmented. Three different datasets have been considered with different sizes and characteristics (4–6). The main achievement of this approach is the high accuracy of classification with simple implementation.

## 2. Related work

Several machine and deep learning algorithms have been proposed for detecting brain tumors from magnetic resonance and CT images. Several findings confirm the importance of MRI and image processing tools to identify brain tumors. The MRI scanners are used to create images of organs in the body, for cases such as fractures, bone dislocations, lung infections, pneumonia, and COVID-19.

Sindhumol et al. (7) introduced an approach for improving brain tumor classification from magnetic resonance images using spectrum angle-dependent feature extraction and Spectral Clustering Independent Component Analysis (SCICA). The magnetic resonance images are firstly divided into clusters depending on spectral distance. Then, Independent Component Analysis (ICA) is applied on the clustered data. A Support Vector Machine (SVM) is used for the classification process. Rating was done using *T*1 weighted, *T*2 weighted, and proton density fluid inversion recovery images. To determine the stability and effectiveness of SC-ICA-based classification, a comparison with ICA-based SVM and other conventional classifiers was performed. For a recurrent lesion, ICA-based SVM analysis achieves 98% for accuracy. Hemanth et al. (8) introduced a CNN-based automated segmentation approach. This approach comprises pre-processing, average filtering, segmentation, feature extraction, and a Neural Network (NN) for classification. An accuracy of 91% has been attained. Mallick et al. (9) suggested an image compression strategy based on a Deep Wavelet Auto-encoder (DWA). A Deep Neural Network (DNN) is employed in the classification phase. An accuracy of 96% has been acquired.

Anaraki et al. (10) presented an approach for MRI brain tumor identification based on CNNs and Genetic Algorithms (GAs). In addition, an ensemble approach was used to reduce the variation of prediction error. For classifying three glioma grades, an accuracy of 96% was obtained. Nalepa et al. (11) provided an end-to-end classification approach for Dynamic Contrast-Enhanced Magnetic Resonance Imaging (DCE-MRI). This strategy attained a 99% accuracy. Amin et al. (12) presented an automated approach for detecting brain tumors from MRI datasets. For the segmentation of potential lesions, several approaches have been used. For the classification procedure, the SVM classifier was used. It achieved an average accuracy of 98%. Gupta et al. (13) developed a non-invasive approach for tumor identification from T2-weighted MRI. Pre-processing improves the magnetic resonance images, which were then segregated using the multilayer customization of the Otsu thresholding technique. From the segmented image, several textural and form features are recovered, and two dominant ones are chosen using an entropy measure.

Sumitra and Saxena (14) introduced an NN technique for identifying magnetic resonance images for brain. It is divided into three steps: feature extraction, dimensionality reduction, and classification. Using Principal Component Analysis (PCA), important characteristics such as mean, median, variance, and correlation values of maximum and minimum intensity are obtained from magnetic resonance images. An NN is built depending on back-propagation. The classifier classifies images as normal, benign, or malignant based on the category to which they belong. The classification accuracy on a brain imaging testing dataset was 73%. Using GAs and an SVM, Jafari and Shafaghi (15) developed a hybrid approach for categorizing brain tumor tissues in MRI datasets. The introduced system has four stages. Noise reduction and contrast enhancement are done during pre-processing in the first stage. The second stage involves segmentation. Morphological operations are used to remove the skull from the images. The selection and extraction of features is the third stage. The features are classified into four categories: static features, Fourier and wavelet transform histograms, and a mixture of them. The features are chosen using GAs. Finally, the selected features are fed into the SVM classifier, which achieves an accuracy of 83.22% in detecting normal and abnormal activities.

Jayachandran and Dhanasekharan developed a hybrid algorithm for diagnosing brain tumors from magnetic resonance images, based on statistics and SVM classifiers (16). Noise reduction, feature extraction, feature reduction, and classification are the four utilized steps of this algorithm. To reduce noise and prepare the image for feature extraction, the anisotropic filter is used. Using the Gray Level Co-occurrence Matrix (GLCM), the texture features are then extracted. The extracted features are then reduced using PCA. Finally, an SVM classifier is utilized for classification. It yields an accuracy of 95.80%. Selvapandian et al. (17) proposed a Non-Sub-Sampled Contourlet Transform-based (NSCT) method for brain tumor diagnosis. The classification procedure is carried out using the Adaptive Neuro Fuzzy Inference System (ANFIS). After that, morphological functions are used to segment the tumor sections in the glioma brain images.

A learning-based system for robust and automated nucleus segmentation with shape preservation was suggested by Xing et al. (18). Initial deep CNN filtering is followed by iterative region merging segmentation using a selective sparse shape model. It makes use of the benefit of faster computations, making it suitable for real-time applications. This system achieves a sensitivity of 89% and an accuracy of 85%. Narayana and Reddy (19) introduced a median filter GA segmentation technique for the segmentation operation. With an SVM classifier, the GLCM is used including the features. An accuracy of 91.23% has been obtained. Zaw et al. (20) developed an algorithm for detecting tumor locations in distinct brain magnetic resonance images, and predicting whether or not the discovered region is a tumor. Pre-processing, pixel removal, maximum entropy cut-off, statistical feature extraction, and a Naive Bayes classifier are used. The accuracy of this algorithm was 94%. Veeramuthu et al. (21) proposed a Combined Feature and Image-based Classifier (CFIC) for brain tumor classification. This approach was evaluated using the kaggle brain tumor detection 2020 dataset. It has given a sensitivity, a specificity, and an accuracy of 98.86, 97.14, and 98.97%, respectively.

An algorithm for detecting brain tumors was developed by Astina Minz and Chandrakant Mahobiya. It revealed lower error rates and required less training time, but it has a limitation that it can only optimize the margin for features that have previously been described (22). This algorithm achieved an accuracy of 89.90% and a precision of 74%. Raju et al. (23) used Bays scan fuzzy clustering segmentation, information-theoretic scatters, and wavelet features for brain tumor diagnosis. An accuracy of 93% has been achieved. Sert et al. (24) presented Single Image Super Resolution (SISR) and a Maximum Fuzzy Entropy Segmentation (MFES) method for brain tumor detection and segmentation. For feature extraction and classification, the ResNet model and the SVM were employed, respectively. An accuracy of 95% has been obtained. Deepak et al. (25) presented a classification technique for extracting features from brain magnetic resonance images based on transfer learning with GoogLeNet. To categorize the extracted features, the SVM classifier was used. The presented method achieved an accuracy of 98%.

## 3. Materials and methods

### 3.1. Datasets

Three different datasets of MRI brain tumors are used to evaluate the proposed approach. The datasets are briefly described in this section. There are 155 images for tumor cases and 155 images for normal cases for the first MRI brain tumor dataset (4). The second MRI brain tumor dataset (5) includes 1,500 images for tumor cases and 1,500 images for normal subjects. The third MRI brain tumor dataset (6) comprises 5,504 images for tumor cases and 6,159 images for normal subjects.

**Figure 1:**
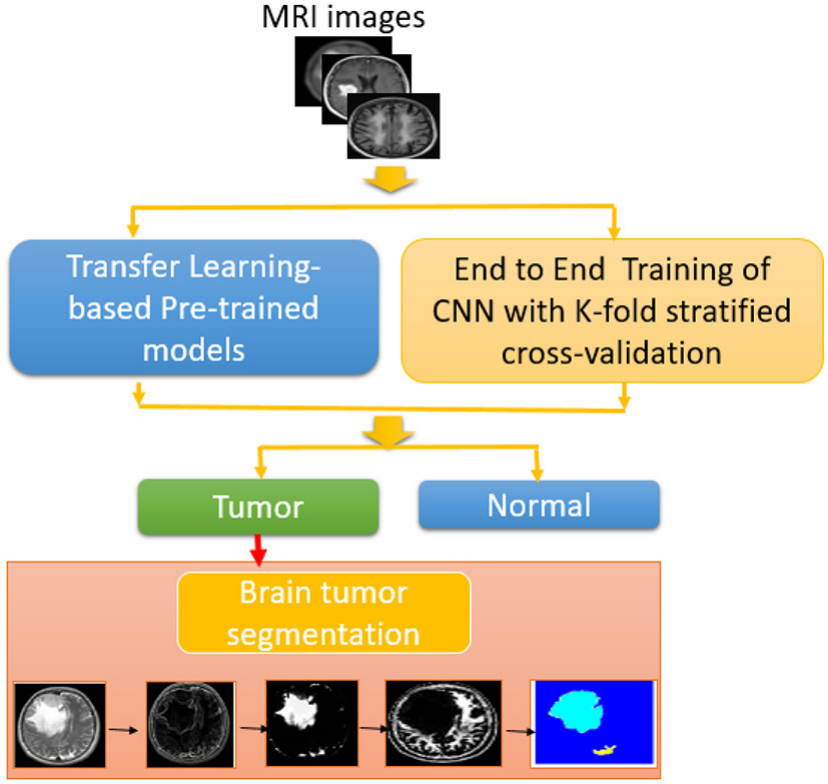
Block diagram of proposed methodology for brain tumor detection and segmentation.

The proposed approach is shown in Figure 1. Magnetic resonance images are used as input to the proposed brain tumor detection approach. Different CNN-based models combined with segmentation techniques, namely, transfer-learning-based models and an end-to-end CNN model have been studied and compared. As previously indicated, the ResNet50, Inceptionv3, and InceptionResNetv2 were used in the transfer-learning-based models. The *k*-fold stratified cross-validation was used to train the BRAIN-TUMOR-net model from scratch. An input layer, three convolutional layers, three Rectified Linear Unit (ReLU) layers, and three Batch Normalization (BN) layers make up the proposed BRAIN-TUMOR-net model structure. For dimensionality reduction, two pooling layers are employed. A Fully-Connected (FC) layer, a softmax layer, and a classification layer are used at the end of the model.

### 3.2. Transfer-learning-based approach

Deep learning from scratch is a time-consuming process that requires data classification and division. Transfer learning is ideal for removing the huge strain of this process. According to the input characteristics, transfer learning causes little modifications in deep pre-trained networks. The used dataset is partitioned into two datasets randomly, with a 75/25 training/testing ratio. The pre-trained models were loaded, and the BN, ReLU, and softmax layers were substituted for the last three FC layers.

### 3.3. State-of-the-art CNNs for transfer learning

Recent CNN models for brain tumor detection are addressed in this section.

- **ResNet**. Deep residual learning network is a new tool for training very deep neural networks. Identity mapping is used for shortcut connections in the deep residual learning network. It is a new way for training very deep neural networks. In a range of computer vision challenges, ResNet exceeded the state-of-the-art networks and won the ImageNet ILSVRC 2015 classification competition.
- **Inceptionv3**. Its architecture is based on Szegedy et al. publication “Rethinking the Inception Architecture for Computer Vision” (2015), which presented an improvement to the inception module to enhance ImageNet classification accuracy, dramatically (26). The authors proposed Inceptionv2 and Inceptionv3 (27). Factorization, which breaks convolutions into smaller convolutions and other minor adjustments to Inceptionv1, were introduced in Inceptionv2. The typical 7 × 7 convolution has been factored into three 3 × 3 convolutions. However, Inceptionv3 is a version of Inceptionv2 that includes a BN-auxiliary. The BN-auxiliary refers to the variant in which the fully-linked layer of the auxiliary classifier, rather than merely convolutions, is normalized. The model [Inceptionv2 + BN-auxiliary] is referred to as Inceptionv3. The Inception module reduces the grid size, which expands the filter banks.
- **InceptionResNetv2**. It is a convolutional neural architecture that uses residual connections from Inception designs. The residual connection takes the place of the filter concatenation stage (28). This network is able to classify 1000 categories.

The dataset is divided into three parts randomly, with the ratio of 75/25 for training or validation/testing. After loading the pre-trained models, the last three fully-connected layers were replaced with BN, ReLU, and softmax layers. The training approaches used in this paper demonstrated their ability to control the degradation problem, while also providing the required convergence in a short time. Due to its high convergence and short running duration, Stochastic Gradient Descent (SGD) is employed for training (29). The ReLU is used to activate all convolutional layers. The main objective of the proposed approach is to combine image batch identification with a fine-tuned classifier to classify many instances as tumor or normal cases (30).

### 3.4. Convolutional neural network trained from scratch

Deep learning models have been employed in a variety of medical data classification, segmentation, and lesion detection applications. Medical imaging techniques such as MRI, X-ray, and CT are used to generate medical images. Machine learning and deep learning models may be evaluated on MRI, CT, and X-ray datasets. This paper provides a number of CNN-based deep learning models for identifying tumor instances by categorizing magnetic resonance images as normal or tumor cases (31–34).

**Figure 2:**
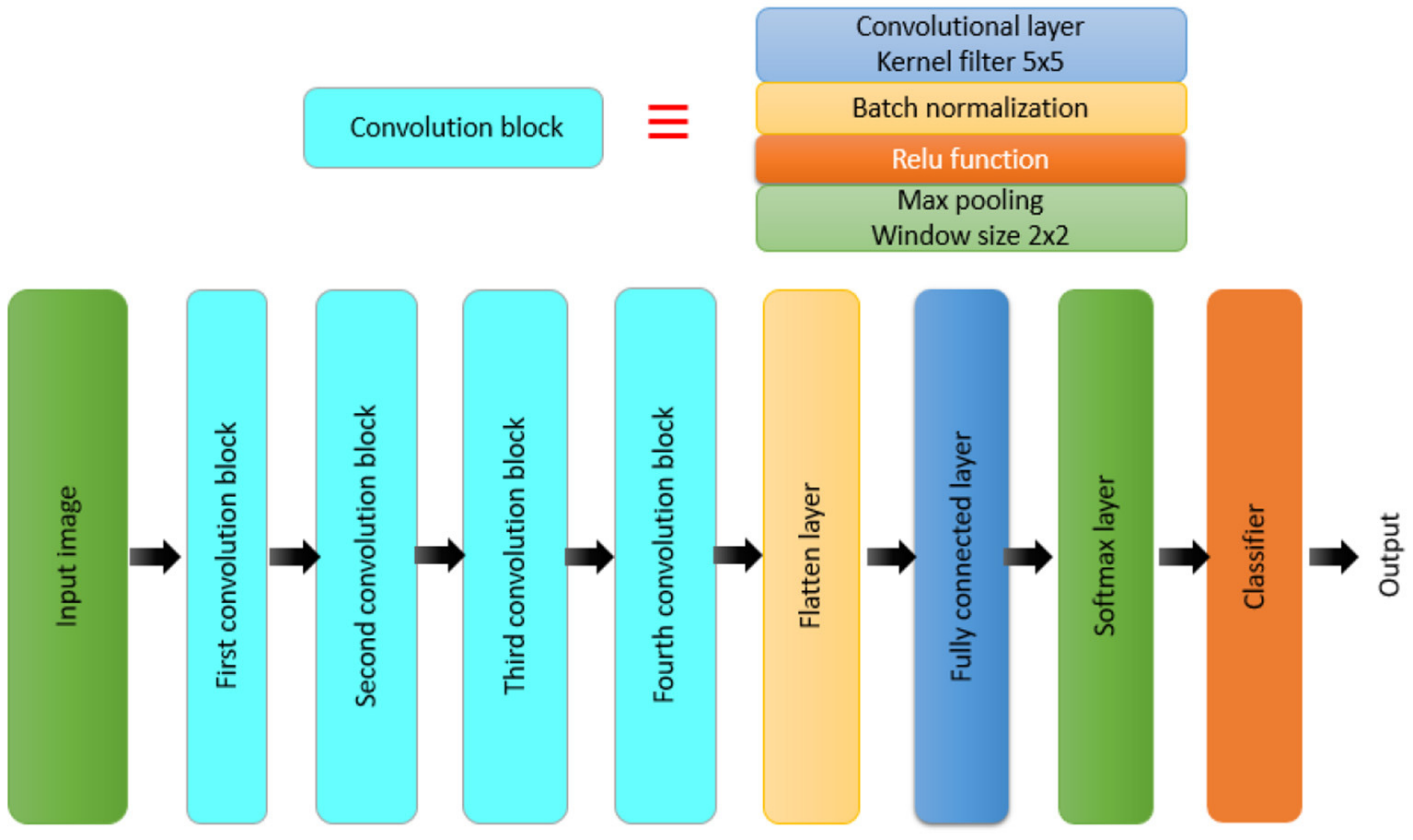
Block diagram for the BRAIN-TUMOR-net model.

Furthermore, a model is built from scratch for the classification task. Figure 2 presents the structure of the BRAIN-TUMOR-net.

A CNN model is made up of several layers, including an input layer, convolutional layers, pooling layers, FC layers, and an output layer (32, 33, 35–38). The proposed BRAIN-TUMOR-net is constructed as follows:

- Input layer. The inputs are magnetic resonance images with a resolution of 224 × 224 pixels.
- COVN layers. The convolutional layer (Conv), the BN layer, and the ReLU layer make up the COVN layers. The convolutional layer captures and compresses image features to create feature maps. As a consequence, convolutions were conducted over the input images for the first, second, third, fourth, and fifth Conv layers, with different filters (8, 16, 32, 64, and 128) and a fixed window size of 3. The BN layers are used in optimization to reduce overfitting and improve test accuracy. The activations of the preceding layer are normalized for each batch during training. To incorporate element-wise non-linearity, a ReLU activation function is used.
- Pooling layer. This layer is used to extract the most important features from each feature map. We use the max-pooling method for pooling operations. The max-pooling layer vectors are concatenated to form a fixed-length feature vector. The stride is set to 2 and the max-pooling window is set to 2 × 2.
- Fully-Connected (FC) layer. It takes a simple vector as input and returns a single vector as output. The proposed model has four FC levels. The last layer is an FC output layer with softmax activation for classifying the input images into two categories.

The principal structural components of a CNN network are convolution, BN, and pooling layers. The convolution layers extract the local features, and the BN layers normalize them. Pooling layers are used to minimize the number of extracted features. To reflect fluctuations in local activity levels, max-pooling is used. It displays the edges wih considerable details. The highest values obtained are mostly associated with edges. Magnetic resonance images are rich in details. The resulting feature map can be represented as follows:


(1) $$\begin{array}{l} {Y_{j}}^{l}=f\left(\underset{i\in N_{j}}{\sum }{Y_{i}}^{l-1}*{X_{ij}}^{l}+{b_{j}}^{l}\right) \end{array}$$

where ${Y_{j}}^{l}$ represents the local features obtained from the previous layer, ${X_{ij}}^{l}$ represents the adjustable kernels. In order to prevent the overfitting, the bias is used and denoted by ${b_{j}}^{l}$. The pooling process is implemented as follows:


(2) $$\begin{array}{l} {Y_{j}}^{l}=down\left({Y_{j}}^{l-1}\right) \end{array}$$

where *down*(.) represents the down-sampling function. The FC layers have full connections to all activations in the previous layer. The FC layer provides discriminative features for the classification of the input image into various classes.

Whenever a classification task, whether binary or multi-class classification, is performed, the data is divided into train and test sets, and the model is trained to improve the accuracy (39). Numerous performance metrics and data splitting mechanisms are becoming increasingly important (40). As a result, stratified *k*-folds and a variety of performance measures may be employed to help in the development of a reliable deep-learning-based model (41). The model precise accuracy cannot be determined, since the model accuracy is altered by modifying the random state values. It samples the data without consideration of class distributions. In case of binary classification, and out of a 100% dataset, 80% belong to class 0 and the remainder to class 1. Through the utilization of random sampling to achieve this balance, there is a strong chance to have different class distributions between training and testing. Tearing on such a dataset will result in inaccurate results.

The most popular validation technique is the *k*-fold technique. The division of the training dataset into *k*-folds is known as cross-validation. The first *k* − 1 folds are used for training, while the remaining fold is used for testing. This process is repeated for each fold. *k* folds are fitted and evaluated collectively, and the mean accuracy for all of these folds is returned. This technique produced promising results for balanced classification issues, but it did not work for imbalanced classes. This is because cross-validation randomly divides the data without taking into consideration class imbalance. As a result, rather than splitting the data randomly, the solution is to stratify it. The stratified *k*-fold cross-validation technique is a variation of the cross-validation commonly used for classification issues. It maintains the same class ratio as in the original dataset throughout the *k*-fold technique. So, by using a stratified *k*-fold technique, the same class ratio may be maintained throughout all *k* folds (42). The essential configuration option for *k*-fold cross-validation is the number of folds *k* (43). When the number *k* is set too high, the bias of the actual error rate estimator becomes minimal, but the estimator variance and time consumption become large. If *k* is small, the calculation time decreases, and the estimator variance decreases, but the estimator bias increases (44). The most common values are *k* = 3, *k* = 5, and *k* = 10. As a consequence, if a maximum classification accuracy is required, the ideal value of *k* needs to be chosen. The value of *k* in this paper is set to 5.

### 3.5. MRI brain tumor segmentation approach

The pixel value properties of the MRI datasets are used to segment the data. On the MRI datasets, the following steps were used in the segmentation process:

1. Post-processing:
  - Image enhancement.
  - Utilization of the usual shrink denoising process to remove noise from the images.
  - Edge preservation by applying a bilateral filter method on the denoised output.
2. Segmentation:
  - Edge-based segmentation using a 3 × 3 mask and the Kirsch operator. A 3 × 3 mask is utilized to implement the Sobel operator. Also, a 5 × 5 mask is utilized to build the sophisticated Sobel operator.
  - Magnetic resonance brain image segmentation based on thresholds. The Otsu threshold algorithm is implemented.
  - Clustering-based segmentation. The *k*-means clustering method with a predetermined number of iterations and a certain value of *k* was developed. The adaptive *k*-means clustering technique was used. The number of iterations to convergence has been determined. In addition, the fuzzy *c*-means clustering technique was used.
  - Watershed algorithm with marker control. The segmentation function was accomplished using the Watershed technique with gradient magnitude.

### 3.6. Performance metrics

The proposed approach performance is assessed using conventional metrics such as sensitivity (SEN), specificity (SPEC), accuracy (ACC), precision (PRECI), Matthews Correlation Coefficient (MCC), *F*1_*score*, kappa, and false positive rate (*F*_*pr*_). The number of successfully detected anomalous cases (*T*_*p*_) is the true positive. The number of accurately detected normal instances (*T*_*n*_) is the true negative. A false positive (*F*_*p*_) is a collection of normal instances designated as anomaly diagnoses. A false negative (*F*_*n*_) is a collection of abnormalities seen as normal (45, 46).

Sensitivity is given as:


(3) $$\begin{array}{l} SEN=\frac{T_{p}}{T_{p}+F_{n}}\times 100 \end{array}$$

Specificity is given as:


(4) $$\begin{array}{l} SPE=\frac{T_{n}}{T_{n}+F_{p}}\times 100 \end{array}$$

Accuracy is given as:


(5) $$\begin{array}{l} ACC=\frac{T_{p}+T_{n}}{T_{p}+T_{n}+F_{p}+F_{n}}\times 100 \end{array}$$

Precision is given as:


(6) $$\begin{array}{l} PRECI=\frac{T_{p}}{T_{p}+F_{p}} \end{array}$$

Matthews correlation coefficient (MCC) is defined as:


(7) $$\begin{array}{l} MCC=\frac{T_{p}\times T_{n}-F_{p}\times F_{n}}{\sqrt{\left(T_{p}+F_{p}\right)\times \left(T_{p}+F_{n}\right)\times \left(T_{n}+F_{p}\right)\times \left(T_{n}+F_{n}\right)}}\\ \times 100 \end{array}$$

False positive rate is given as:


(8) $$\begin{array}{l} F_{pr}=\frac{F_{p}}{T_{n}+F_{p}} \end{array}$$

*F*1_*score* is given as:


(9) $$\begin{array}{l} F1\text{\_}score=\frac{T_{p}}{T_{p}+\frac{1}{2}\left(F_{p}+F_{n}\right)}\times 100 \end{array}$$

Kappa coefficient is defined as:


(10) $$\begin{array}{l} kappa=\frac{2\times \left(T_{p}\times T_{n}-F_{n}\times F_{p}\right)}{\left(T_{p}+F_{p}\right)\times \left(F_{p}+T_{n}\right)\times \left(T_{p}+F_{n}\right)\times \left(F_{n}+T_{n}\right)}\\ \;\times 100 \end{array}$$

## 4. Simulation results

The proposed approach is evaluated on three different publicly-available datasets. The performance of the CNN models differs from one dataset to another according to the size of the dataset.

### 4.1. Results on the first MRI dataset

**Figure 3:**
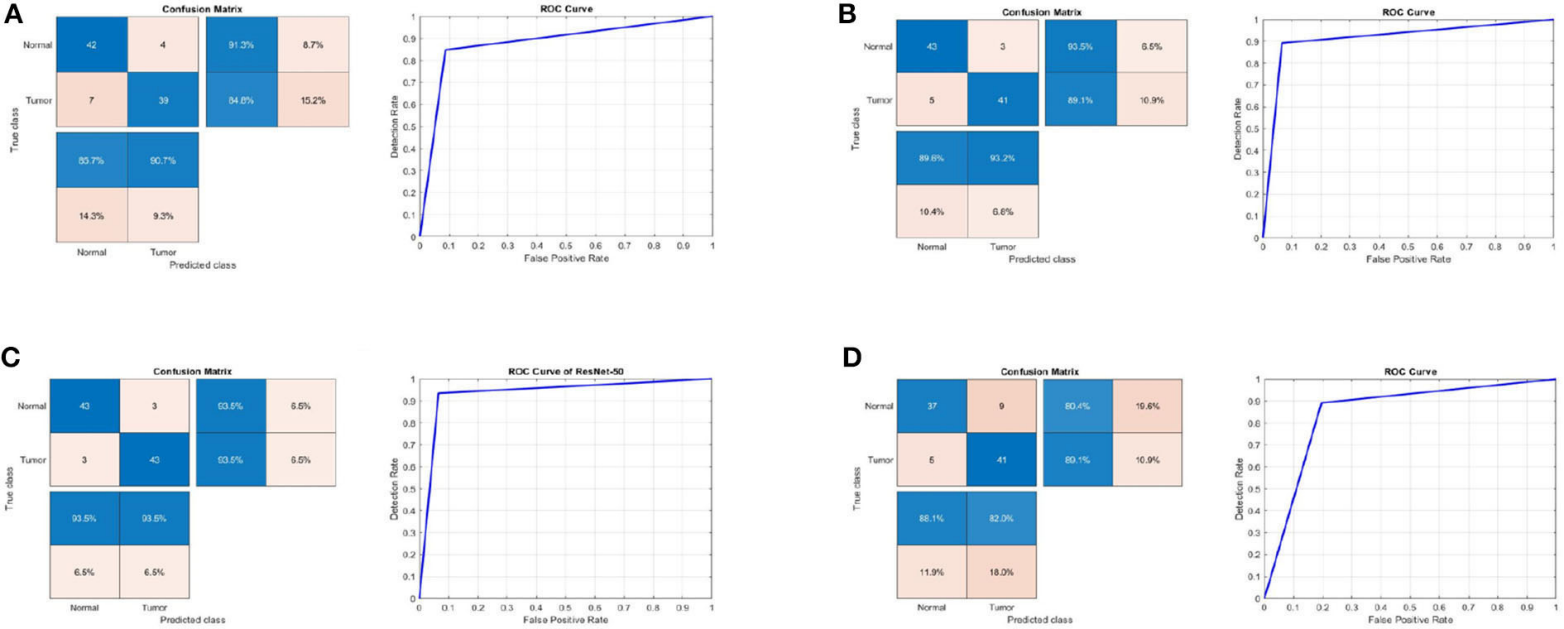
Confusion matrix and ROC curve for **(A)** Inceptionv3, **(B)** InceptionResNetv2, **(C)** ResNet50, and **(D)** BRAIN-TUMOR-net on the first dataset.

Table 1 summarizes the results for the proposed approach in terms of SEN, SPEC, ACC, PRECI, MCC, *F*_*pr*_, *F*1_*score*, kappa, and error using three transfer-learning-based CNN models and a CNN model trained from scratch on the first MRI dataset. Figure 3 presents the receiver operating characteristic (ROC) curves and confusion matrices for Inceptionv3, InceptionResNetv2, ResNet50 and BRAIN-TUMOR-net models. It is clear that the ResNet50 model achieves the highest performance among other models on the first MRI dataset. It achieves a sensitivity of 93.48%, a specificity of 93.48%, an accuracy of 93.48%, a precision of 93.48%, an MCC of 86.96%, a false positive rate of 0.0652, an *F*1_*score* of 93.48%, a kappa of 86.96%, and an error of 0.0652.

**Table 1:** Detection performance results for various CNN models with a 75/25 training/testing ratio on the first dataset.

**Models**	**Evaluation metric**								

	**ACC**	**SEN**	**SPE**	**PRECI**	**F1_score**	**MCC**	**Error**	**Kappa**	**FPR**
InceptionResNetv2	0.9130	0.9348	0.8913	0.8958	0.9149	0.8269	0.0870	0.8261	0.1087
Inceptionv3	0.8804	0.9130	0.8478	0.8571	0.8842	0.7625	0.1196	0.7609	0.1522
ResNet50	0.9348	0.9348	0.9348	0.9348	0.9348	0.8696	0.0652	0.8696	0.0652
Transfer learning model	0.9130	0.9130	0.9130	0.9130	0.9130	0.8261	0.0870	0.8261	0.0870
BRAIN-TUMOR-net	0.8478	0.8043	0.8913	0.8810	0.8409	0.6983	0.1522	0.6957	0.1087

### 4.2. Results on the second MRI dataset

**Figure 4:**
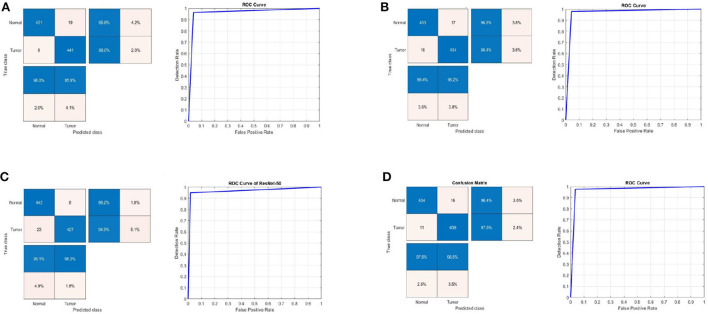
Confusion matrix and ROC curve for **(A)** Inceptionv3, **(B)** InceptionResNetv2, **(C)** ResNet50, and **(D)** BRAIN-TUMOR-net on the second dataset.

Table 2 shows the detection performance results for different CNN models with a 75/25 training/testing ratio on the second dataset. Figure 4 presents the ROC curves and confusion matrices for Inceptionv3, InceptionResNetv2, ResNet50 and BRAIN-TUMOR-net models, respectively. It is clear from the obtained results that the BRAIN-TUMOR-net model achieves the highest performance among the other proposed ones on the second MRI dataset. It achieves a sensitivity of 96.44%, a specificity of 97.56%, an accuracy 97%, a precision of 97.53%, an MCC of 94.01%, a false positive rate of 0.0244, an *F*1_*score* of 96.98%, a kappa of 94%, and an error of 0.0300.

**Table 2:** Detection performance results for the proposed CNN models with a 75/25 training/testing ratio on the second dataset.

**Models**	**Evaluation metrics**								

	**ACC**	**SEN**	**SPE**	**PRECI**	**F1_score**	**MCC**	**Error**	**Kappa**	**FPR**
InceptionResNetv2	0.9689	0.9578	0.9800	0.9795	0.9685	0.9380	0.0311	0.9378	0.0200
Inceptionv3	0.9633	0.9622	0.9644	0.9644	0.9633	0.9267	0.0367	0.9267	0.0356
ResNet50	0.9656	0.9822	0.9489	0.9505	0.9661	0.9316	0.0344	0.9311	0.0511
BRAIN-TUMOR-net	0.9700	0.9644	0.9756	0.9753	0.9698	0.9401	0.0300	0.9400	0.0244

### 4.3. Results on the third MRI dataset

**Figure 5:**
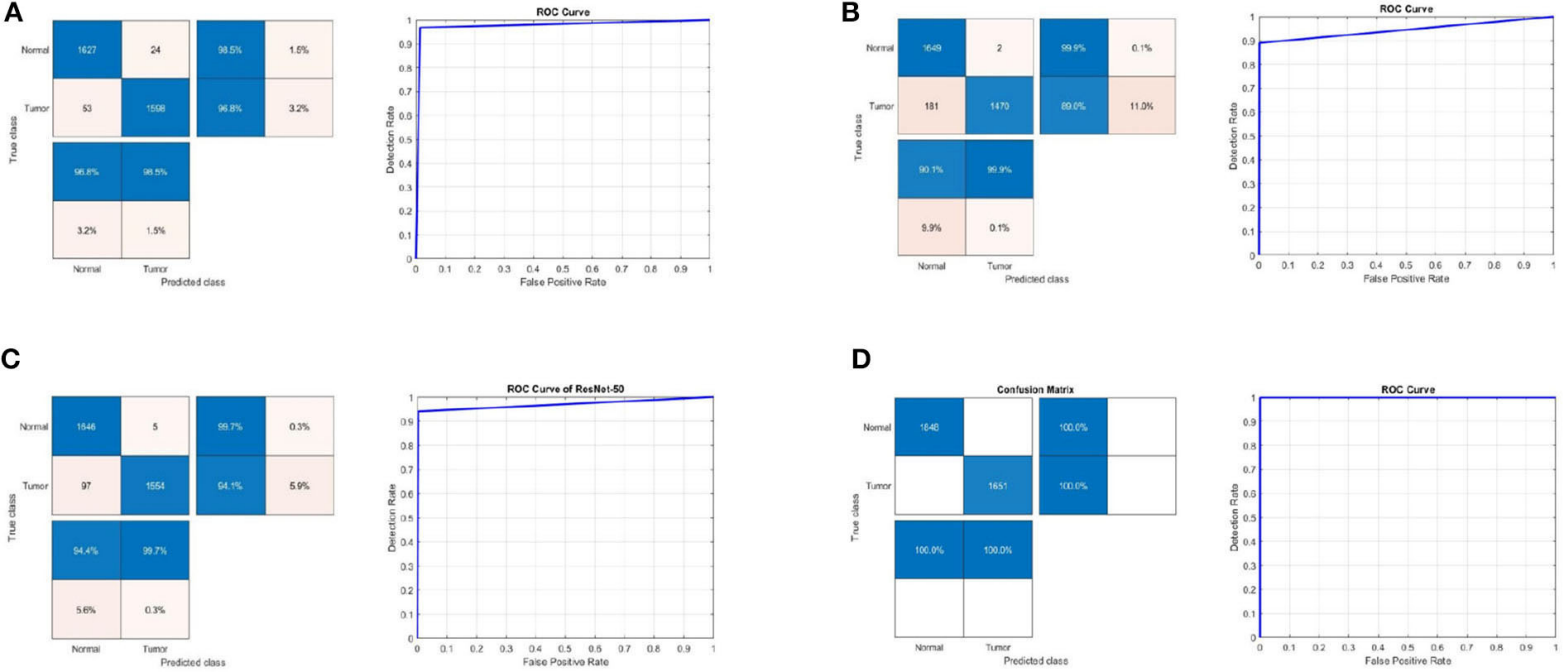
Confusion matrix and ROC curve for **(A)** Inceptionv3, **(B)** InceptionResNetv2, **(C)** ResNet50, and **(D)** BRAIN-TUMOR-net on the third dataset.

Table 3 shows the detection performance results for different CNN models with a 75/25 training/testing ratio on the third dataset. Figure 5 presents the ROC curves and confusion matrices for Inceptionv3, InceptionResNetv2, ResNet50 and BRAIN-TUMOR-net models. It is clear from the obtained results that the BRAIN-TUMOR-net model achieves the highest performance among the other proposed ones on the third MRI dataset. It achieves a sensitivity of 100%, a specificity of 100%, an accuracy 100%, a precision of 100%, an MCC of 100%, a false positive rate of 0.0, an *F*1_*score* of 100%, a kappa of 100%, and an error of 0.0.

**Table 3:** Detection performance results for the proposed CNN models with a 75/25 training/testing ratio on the third dataset.

**Models**	**Evaluation metric**								

	**ACC**	**SEN**	**SPE**	**PRECI**	**F1_score**	**MCC**	**Error**	**Kappa**	**FPR**
InceptionResNetv2	0.9446	0.9988	0.8904	0.9011	0.9474	0.8944	0.0554	0.8892	0.1096
Inceptionv3	0.9767	0.9855	0.9679	0.9685	0.9769	0.9535	0.0233	0.9534	0.0321
ResNet50	0.9691	0.9970	0.9412	0.9443	0.9699	0.9397	0.0309	0.9382	0.0588
BRAIN-TUMOR-net	1.00	1.00	1.00	1.00	1.00	1.00	0.0	1.00	0.0

### 4.4. Results for BRAIN-TUMOR-net based on stratified *k*-fold validation

**Figure 6:**
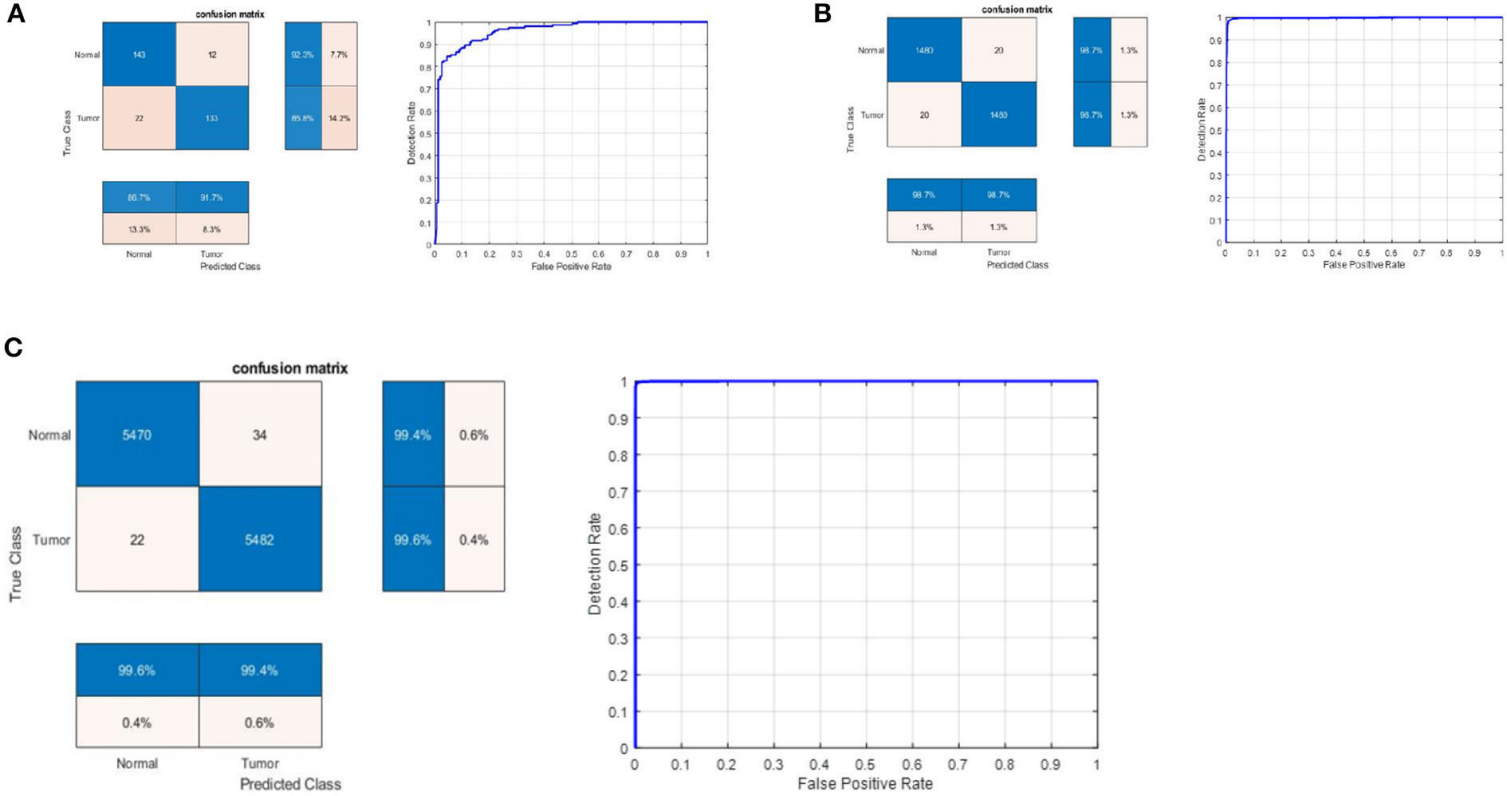
Confusion matrix and ROC curve for BRAIN-TUMOR-net on **(A)** First dataset, **(B)** Second dataset, and **(C)** Third dataset.

**Figure 7:**
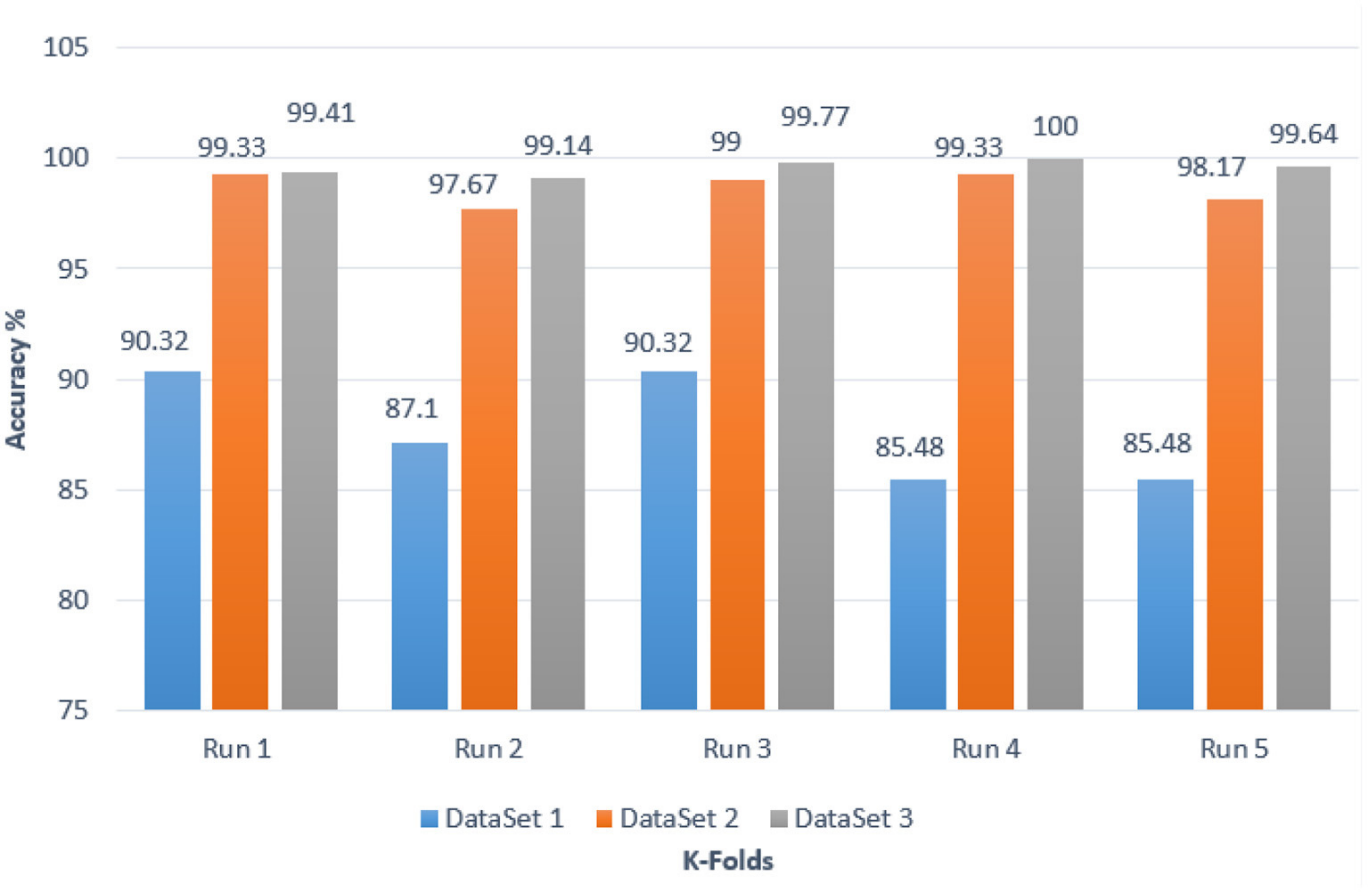
Accuracy values for different *k*-folds using the BRAIN-TUMOR-net with stratified *k*-fold validation on the three MRI brain tumor datasets.

Stratified *k*-fold validation is combined with BRAIN-TUMOR-net model in order to get a more stable error. Table 4 presents the detection performance results from BRAIN-TUMOR-net using a stratified *k*-fold validation model with a 75/25 training/testing ratio on the three MRI datasets. Figure 6 presents the ROC curves and confusion matrices for the first, second and third datasets. Figure 7 provides the value of accuracy for each *k*.

**Table 4:** Detection performance results for BRAIN-TUMOR-net based on stratified *k*-fold cross validation with a 75/25 training/testing ratio on the three datasets.

**Dataset**	**Evaluation metrics**								

	**ACC (%)**	**SEN (%)**	**SPE (%)**	**PRECI (%)**	**F1_score (%)**	**MCC (%)**	**Error**	**Kappa (%)**	**FPR**
First MRI dataset	89.03	92.26	85.81	86.67	89.38	78.23	0.1097	78.06	0.1419
Second MRI dataset	98.67	98.67	98.67	98.67	98.67	97.33	0.0133	97.33	0.0133
Third MRI dataset	99.49	99.38	99.60	99.60	99.49	98.98	0.0051	98.98	0.0040

### 4.5. MRI brain tumor segmentation results

On magnetic resonance brain images, an image segmentation method is used to separate similar sections of the image based on the gray level values of the pixels. The primary goal of segmenting magnetic resonance brain images is to aid in tumor detection. Edge-based segmentation (Krisch and Sobel), threshold-based segmentation (Otsu), and clustering algorithms, namely k-means, adaptive k-means, fuzzy c-means, and marker-controlled watershed, were used as segmentation techniques. Segmentation process consists of seven steps to generate the segmented image. The normal shrink image is generated from the normal shrink denoising algorithm. Edge preservation is achieved by applying a bilateral filter on the denoised output.

**Figure 8:**
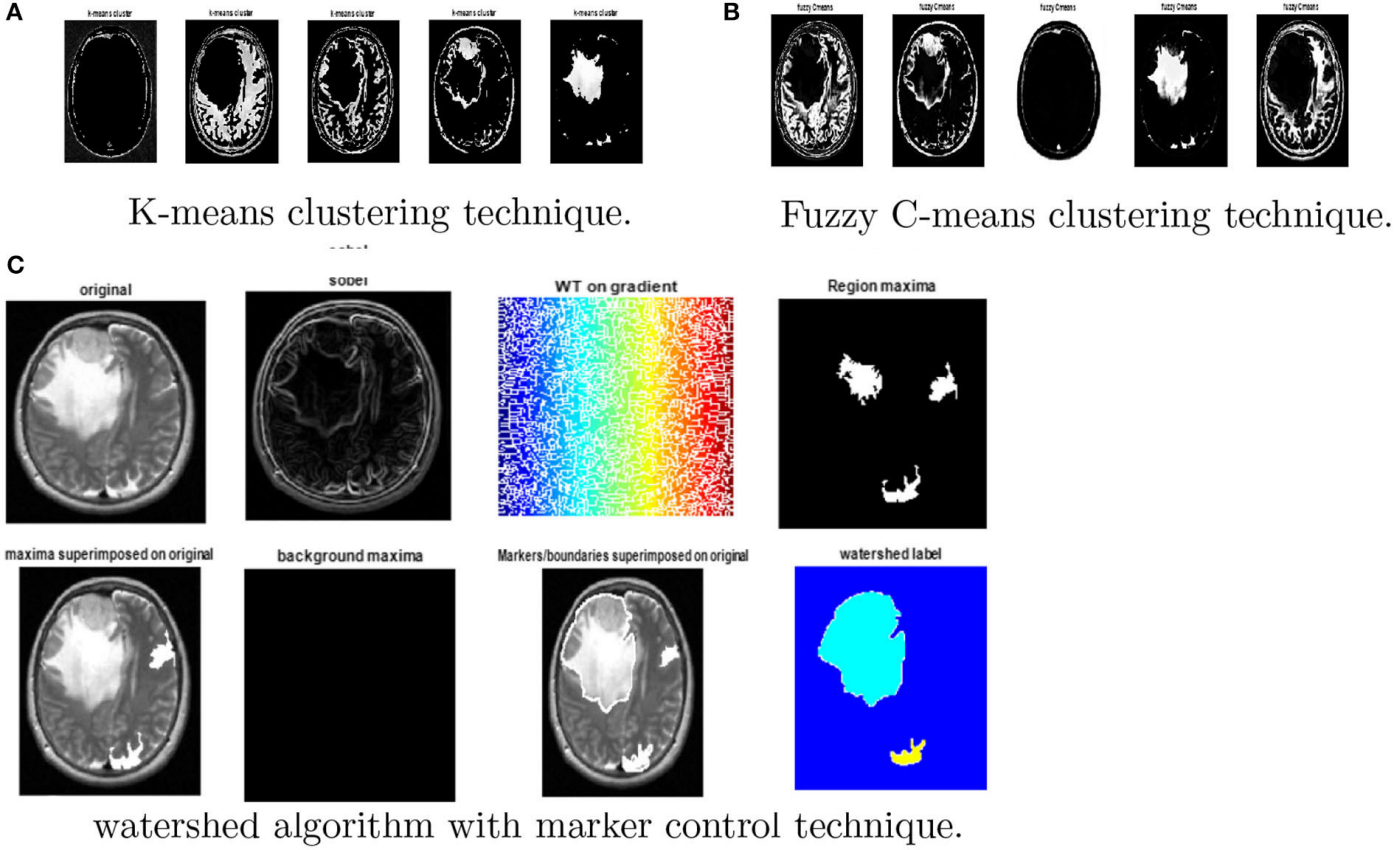
Results obtained from watershed algorithm with marker control technique. **(A)** K-means clustering, **(B)** Fuzzy c-means clustering, and **(C)** watershed algorithm.

Various clustering techniques such as k-means clustering, adaptive clustering, and fuzzy c-means clustering have been implemented. Figure 8 depicts the outcomes for *k*-means clustering, fuzzy *c*-means clustering, and the results obtained from the watershed algorithm with marker control.

Finally, the magnitude of the gradient is determined and employed as a segmentation function. Then, the watershed transform is applied on the gradient magnitude. The original image is then changed into another image by employing morphological operators to iterate with other images of specified shape and size.

## 5. Discussion and comparison with the-state-of-the-art methods

As the dataset size grows, the BRAIN-TUMOR-net trained from scratch outperforms the transfer-learning-based techniques. However, the results show that even when using a small dataset, transfer learning produces satisfactory outcomes. State-of-the-art models were trained on 25 million images. Their convolution layer filters were chosen, because they are effective in novel applications like brain tumor detection. Furthermore, the accuracy of the classification application is influenced by the depth of the CNN models.

**Figure 9:**
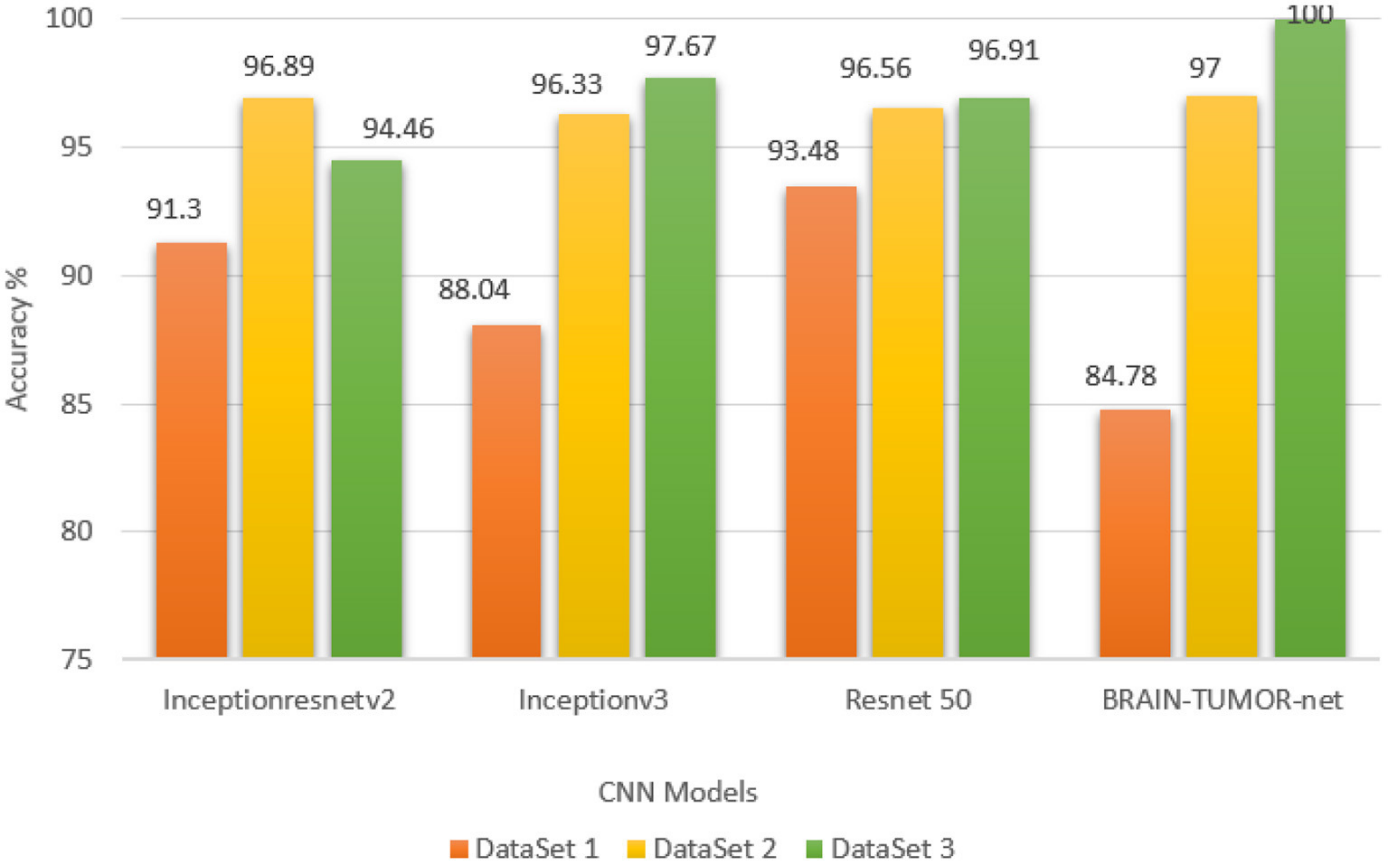
Accuracy values for different CNN models on the three MRI brain tumor datasets.

The stratified *k*-fold validation procedure with 5 folds is employed to get a more steady error rate. This is attributed to the stratified *k*-fold cross-validation capacity to cope with imbalanced data. It keeps the same class ratio as that of the original dataset throughout the *k* folds. The accuracy results for different CNN models on the three MRI brain tumor datasets are shown in Figure 9. The results clearly vary based on the depth of the CNN model, the classification complexity, and the amount of data.

The computation time is the most important metric for comparing various techniques. It is obvious from Table 5 that the ResNet50 model yields run times of 4.68, 9.44, and 24.67 s on the first, second, and third datasets, respectively, which are the shortest times. The Inceptionv3 model was reported to give the second-best runtimes of 7.67, 23.91, and 71.835 s on the first, second, and third datasets, respectively. However, the CNN model trained from scratch using *k*-fold validation has the longest runtimes of 376, 3418, and 16284.818 s on the first, second, and third datasets, respectively. It obtained a level of accuracy of 100%. In the future work, numerous solutions such as image downsizing, adjusting the number of max-pooling layers, and dropout will be investigated to minimize the computation time.

**Table 5:** Computational times of the examined approaches.

**MRI dataset**	**CNN model**	**Elapsed time (s)**
Dataset 1	InceptionResNetv2	31.66
	Inceptionv3	7.67
	ResNet50	4.68
	BRAIN-TUMOR-net	53
	BRAIN-TUMOR-net with K-fold	376
Dataset 2	InceptionResNetv2	68.95
	Inceptionv3	23.91
	ResNet50	9.44
	BRAIN-TUMOR-net	1,069.2
	BRAIN-TUMOR-net with *k*-fold cross validation	3418
Dataset 3	InceptionResNetv2	210.0371
	Inceptionv3	71.83508
	ResNet50	24.6754
	BRAIN-TUMOR-net	11,702.6
	BRAIN-TUMOR-net with *k*-fold cross validation	16284.818355

The proposed approach yields an accuracy level of 100%, which is greater than the levels of the traditional approaches shown in Table 6. These findings support the CNN model ability to execute the essential classification task after being trained from scratch.

**Table 6:** Comparison of the proposed work with state-of-the-art models.

**References**		**Method**	**Accuracy**
Sindhumol et al. (7)		Spectral angle-based feature extraction method and Spectral Clustering Independent Component Analysis (SC-ICA)	98% for SVM and 96.1% for reproduced lesion
Sumitra and Saxena (14)		PCA, Back-propagation neural network	73%
Jafari and Shafaghi (15)		Genetic Algorithm (GA) and Support Vector Machine (SVM),	83.22%
Jayachandran and Dhanasekaran (16)		Statistical, SVM classifier and PCA	95.80%
Xing et al. (18)		The HySIME algorithm initial filtering by deep CNN followed by iterative region merging segmentation by selective sparse shape model.	85%
Zaw et al. (20)		Morphological operation, pixel subtraction, and maximum entropy threshold segmentation with Naive Bayes classifier.	94 %
Narayana and Reddy (19)		Median filter GA segmentation with SVM classifier.	91.23%
Minz and Mahobiya (22)		GLCM (Gray Level Co-occurrence Matrix) for classification boosting.	89.90% & 74.00%
Raju et al. (23)		Bayesian fuzzy clustering segmentation with HSC-based multi SVNN classification method.	93%
Sert et al. (24)		Single image super-resolution for image enhancement and segmentation with maximum fuzzy entropy (MFE) and SVM classifier.	95%
Deepak and Ameer (25)		Min-max normalization, with SVM and KNN classifier.	97.8% & 98%
Hemanth et al. (8)		Average filter, and pixel subtraction with CNN-linkNet classifier.	91%
Mallick et al. (9)		DICOM image processing, and DWT-DNN features with MLP classifier.	96%
Selvapandian and Manivannan (17)		NSCT image enhancement, and GLCM texture features with ANFIS classifier.	98.5%
Nalepa et al. (11)		Sharpening and smoothing filters, threshold segmentation, and SGLD features with ANN classifier.	99%
Anaraki et al. (10)		Image rescaling, and data augmentation with CNN classifier.	96%
Amin et al. (12)		Skull stripping-BSE Gaussian filtering, *k*-Means clustering with SVM classifier.	98%
Gupta and Khanna (13)		Image enhancement-DSR-AD, and Otsu segmentation with SVM classifier.	98%
**Proposed work**			
Dataset 1	InceptionResNetv2	InceptionResNetv2	91.30%
	Inceptionv3	Inceptionv3	88.04%
	ResNet50	ResNet50	93.48%
	BRAIN-TUMOR-net	BRAIN-TUMOR-net	84.78%
	*k*-fold model	*k*-fold validation model	89.03%
Dataset 2	InceptionResNetv2	InceptionResNetv2	96.89%
	Inceptionv3	Inceptionv3	96.33%
	ResNet50	ResNet50	96.56%
	BRAIN-TUMOR-net	BRAIN-TUMOR-net	97%
	*k*-fold Model	*k*-fold validation model	98.67%
Dataset 3	InceptionResNetv2	InceptionResNetv2	94.46%
	Inceptionv3	Inceptionv3	97.67%
	ResNet50	ResNet50	96.91%
	BRAIN-TUMOR-net	BRAIN-TUMOR-net	100%
	*k*-fold Model	*k*-fold validation model	99.49%

## 6. Conclusions

The CNN is regarded as one of the most effective tools for classifying image datasets. It produces the forecast by reducing the image into features without losing the necessary information to make the prediction, correctly. In this paper, three different deep learning models for brain tumor classification have been introduced. Transfer-learning-based models, as well as a CNN model, BRAIN-TUMOR-net, and a model trained from scratch, have been introduced. Three publicly available MRI datasets have been used to test the proposed models. The results show that the BRAIN-TUMOR-net achieves the highest accuracy among the other models as the dataset size increases. It achieves a 100% accuracy on the third dataset, while it achieves 97% and 84.78% accuracy levels on the second and first MRI datasets, respectively. When compared to existing pre-trained models, the proposed model needs extremely less processing power and achieves far higher accuracy outcomes. In future research, optimization techniques can be applied so as to decide the number of layers and filters that can used in the model.

## Data availability statement

The datasets presented in this study can be found in online repositories. The names of the repository/repositories and accession number(s) can be found below: https://www.kaggle.com/ahmedhamada0/brain-tumor-detection?select=yes; https://www.kaggle.com/navoneel/brain-mri-images-for-brain-tumor-detection; https://www.kaggle.com/leaderandpiller/brain-tumor-segmentation.

## Author contributions

FA: study conception and design. MS and HE: data collection. MS, KA, and FT: analysis and interpretation of results. MH, HE, and MS: draft manuscript preparation. All authors reviewed the results and approved the final version of the manuscript.

## Conflict of interest

The authors declare that the research was conducted in the absence of any commercial or financial relationships that could be construed as a potential conflict of interest.

## Publisher's note

All claims expressed in this article are solely those of the authors and do not necessarily represent those of their affiliated organizations, or those of the publisher, the editors and the reviewers. Any product that may be evaluated in this article, or claim that may be made by its manufacturer, is not guaranteed or endorsed by the publisher.
